# Prohibitin is associated with antioxidative protection in hypoxia/reoxygenation-induced renal tubular epithelial cell injury

**DOI:** 10.1038/srep03123

**Published:** 2013-11-04

**Authors:** Tian-Biao Zhou, Yuan-Han Qin, Feng-Ying Lei, Wei-Fang Huang, Gregor P. C. Drummen

**Affiliations:** 1 Department of Pediatric Nephrology, the First Affiliated Hospital of GuangXi Medical University, NanNing 530021, China; 2 Department of Nephrology, the Sixth Affiliated Hospital of Sun Yat-Sen University, Guangzhou, China; 3 Cellular Stress and Ageing Program, Bionanoscience and Bio-Imaging Program, Bio&Nano-Solutions, Helmutstr. 3A, 40472 Düsseldorf, Germany; 4 These authors contributed equally to this work.

## Abstract

Prohibitin is an evolutionary conserved and pleiotropic protein that has been implicated in various cellular functions, including proliferation, tumour suppression, apoptosis, transcription, and mitochondrial protein folding. We recently demonstrated that prohibitin downregulation results in increased renal interstitial fibrosis. Here we investigated the role of oxidative stress and prohibitin expression in a hypoxia/reoxygenation injury system in renal tubular epithelial cells with lentivirus-based delivery vectors to knockdown or overexpress prohibitin. Our results show that increased prohibitin expression was negatively correlated with reactive oxygen species, malon dialdehyde, transforming-growth-factor-β1, collagen-IV, fibronectin, and apoptosis (*r* = −0.895, −0.764, −0.798, −0.826, −0.817, −0.735; each *P* < 0.01), but positively correlated with superoxide dismutase, glutathione and mitochondrial membrane potential (*r* = 0.807, 0.815, 0.739; each *P* < 0.01). We postulate that prohibitin acts as a positive regulator of mechanisms that counteract oxidative stress and extracellular matrix accumulation and therefore has an antioxidative effect.

Renal tubular epithelial cells (RTEC) are polarized cells that constitute the predominant cell type within the tubulointerstitium, and play a critical role in normal kidney function^1^,^2^. Oxidatively induced injury in RTEC cells is accompanied by an increase in transforming growth factor β1 (TGF-β1) and the accumulation of extracellular matrix (ECM), and induces the progression of renal interstitial fibrosis (RIF)^3^,^4^. Injury to RTEC cells has recently been proposed to directly contribute to the accumulation of myofibroblasts in renal tubulointerstitial fibrosis^5^.

Oxidative damage plays an important role in renal diseases and can occur in the kidney through mechanisms that disturb the balance between the production of reactive oxygen species (ROS) and antioxidant defences. This balance determines the overall redox state of the cell, and an increase in the production of ROS or a reduction in the antioxidant capacity induces progressive damage to cells and tissues, and ultimately organ dysfunction^6^.

Prohibitin (PHB) is a multifunctional intracellular protein that plays an important role in the regulation of cell proliferation, apoptosis, and differentiation^7^. It is ubiquitously expressed in a variety of cell types and multiple cellular compartments, including the mitochondria, the nucleus, and the plasma membrane^8^. Several recent lines of research also suggest that PHB plays a crucial role in the oxidative balance of tissues^9^. In our previous studies, we found that suppression of PHB expression was associated with cell apoptosis and RIF progression in a RIF rat model^10^. However, it was difficult to determine whether PHB should be considered a protective or a RIF risk factor. There is some inconsistency in the literature regarding the role of PHB in oxidative injury, primarily because of different assessment methods and because, depending on the cell type or tissue, PHB might be involved in different or multiple processes^11^. To the best of our knowledge, no study is available that reports on the relationship between PHB, oxidative stress, and the pathogenesis of RTEC injury. Therefore, this study was performed to explore what role the expression of PHB plays in hypoxia/reperfusion-induced RTEC injury. It is expected that the insight gained might contribute to a better understanding of the basic mechanisms underpinning RIF and ultimately provide the basis for novel therapeutic interventions to counteract RIF in human patients.

## Results

### General approach

The cells were divided into five groups: (i) normoxic RTEC control (control), (ii) untransduced cells subjected to hypoxia/reoxygenation (H-R), (iii) cells transduced with lentivirus carrying PHB-siRNA subjected to H-R (H-R PHB^–^), (iv) cells transduced with lentivirus carrying *Phb* subjected to H-R (H-R PHB^+^), and (v) cells transduced with control viruses subjected to H-R (H-R negative control). Hypoxia/reoxygenation was chosen, because this method is a physiologically relevant method to experimentally induce endogenous oxidative stress, and hypoxia is a major mechanism in renal interstitial fibrosis. We previously showed that next to near ischemic conditions, i.e., ischemic hypoxia, reestablishment of the energy metabolism by restoring nutrient flow is essential to induce oxidative stress and lipid peroxidation^12^,^13^, and therefore Bhogal's method was adopted^14^.

### Microscopic evaluation of cellular morphologic changes in RTEC

**Figure 1:**
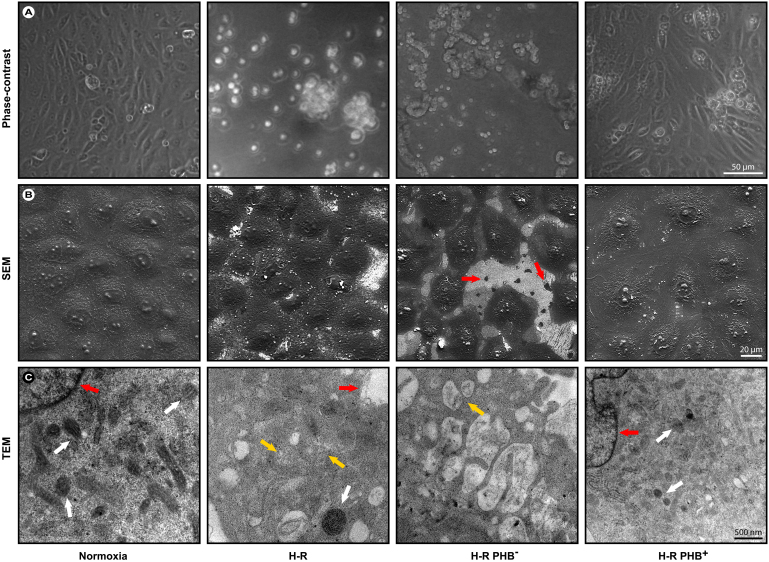
Optical and electron microscopic evaluation of changes in cellular morphology. (A) Phase-contrast, (B) scanning electron microscopy (red arrows indicate cell fragments), and (C) transmission electron microscopic evaluation of mitochondrial changes. Note in the TEM images that there is an increased loss in mitochondrial integrity in the PHB^–^ group compared with the H-R group (yellow arrows) compared with normoxic control cells (white arrows). The damage to mitochondria in the PHB^+^ was alleviated compared with the H-R group (white arrow). Red arrows indicate differences between sharp and contrast-rich membranes and the blebbed and diffuse membranes in the H-R group. H-R group: cell injury induced by hypoxia reoxygenation; H-R PHB^–^: group lentivirus carrying siRNA PHB; H-R PHB^+^ group: lentivirus carrying *Phb*.

An initial morphologic assessment of the cell culture via optical microscopy showed that H-R induces deviation from normal cellular morphology, causes reduction in cell elongation and predominantly rounded, but still partially attached cells were observed (Fig. 1A). These phenomena were significantly aggravated in the H-R PHB^–^ cells, which showed globular morphology, detachment, cell shrinkage, and increased cell clustering. Scanning electron microscopy (SEM) confirmed these initial findings (Fig. 1B) and revealed significant nuclear changes in the reduced PHB (H-R PHB^–^) cells, less coverage of the surface, cellular fragmentation (red arrows) and a general rougher appearance compared with the control or PHB^+^ cells. The latter did not significantly deviate from the normoxic control cells.

To assess the mitochondrial and membrane morphology, transmission electron microscopy was performed. Figure 1C shows normal mitochondria with elongated or round forms and normal cristae (white arrows) under normoxic conditions. Conversely, cells that underwent H-R or additionally had reduced PHB (H-R PHB^–^) showed distinct changes in mitochondrial morphology, i.e., mitochondrial swelling, abnormal cristae, and loss of mitochondrial integrity (yellow arrows). Overall, H-R resulted in a diffuse intracellular appearance with diffuse and ruffled membranes in comparison with normoxic control cells and HR-PHB^+^ cells (red arrows). However, these morphological changes (loss of intracellular integrity) were significantly aggravated in H-R PHB^–^ cells. Conversely, cells in which PHB was overexpressed (HR-PHB^+^) showed similar mitochondrial morphology and intact mitochondria as the normoxic control. There was no significant difference in the morphology between the control viruses and H-R groups (data not shown). Overall, these observations suggested that PHB overexpression was able to counteract the degenerative effects of H-R.

### Expression of prohibitin and extracellular matrix components in RTEC

**Figure 2:**
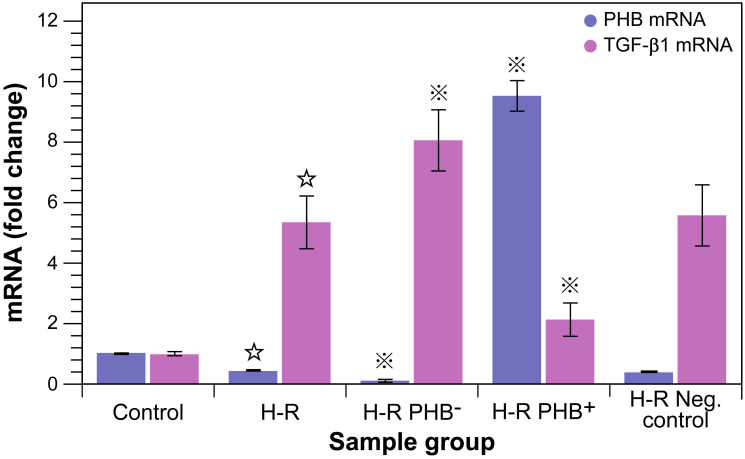
mRNA expression of PHB and TGF-β1 in RTEC. 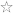
: *P* < 0.01 compared with normal control group, 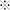: *P* < 0.01 compared with H-R group (cell injury induced by hypoxia reoxygenation group). H-R PHB^–^: transduced with lentivirus carrying siRNA PHB; H-R PHB^+^: transduced with lentivirus carrying *Phb*; H-R negative control: transduced with control viruses.

**Figure 3:**
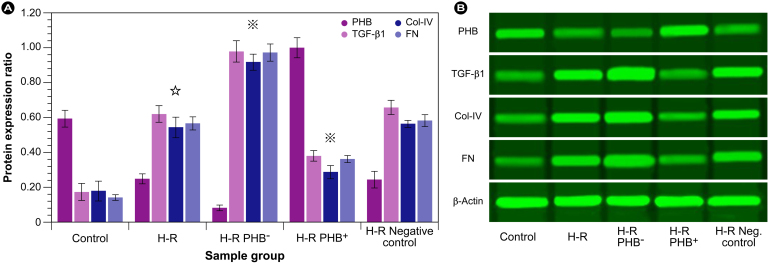
Evaluation of protein expression (Western-blot). (A) Protein ratios were calculated relative to β-actin (loading control). 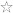: *P* < 0.01 compared with control group, 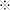: *P* < 0.01 compared with H-R group. (B) Representative Western blot for PHB, TGF-β1, Col-IV, and FN in control, H-R (cell injury induced by hypoxia reoxygenation), H-R PHB^–^ (transduced with lentivirus carrying siRNA PHB), and H-R PHB^+^ transduced with lentivirus carrying *Phb*) groups; H-R negative control: transduced with control viruses. All blots were run under the same experimental conditions and cropped for clarity reasons.

Since microscopic evaluation indicated that modulation of PHB expression affected the extent of the cellular derangements, and PHB overexpression counteracted these phenomena, mRNA (Fig. 2) and protein expression levels (Fig. 3) of PHB and proteins involved in the ECM, i.e., collagen IV (Col-IV) and fibronectin (FN), and the profibrotic factor transforming growth factor beta 1 (TGF-β1), were assessed in the five experimental groups. TGF-β1 was particularly taken into account, since it is regarded to play a central role in interstitial fibrosis, and is reported to affect both pro- and anti-fibrotic signalling, e.g., through the Smad or jagged/notch pathway.

Cells of the H-R group consistently showed a ∼ 2.5 fold lower PHB mRNA expression (∼40% remaining) compared with control (*P* < 0.01; Figure 2), which demonstrates that induction of oxidative stress *per se* reduces the expression of PHB. Equally, TGF-β1 expression was induced as a consequence of H-R, and increased ∼ 5.4 fold compared with normoxic control cells. The desired knockdown of PHB-mRNA (H-R PHB^–^) with the vector was nearly complete as shown in Fig. 2 (∼11% remaining) and overexpression of PHB-mRNA (H-R PHB^+^) was nearly 10 fold higher than control. Compared with H-R only, PHB knockdown under H-R conditions resulted in a further reduction by a factor of ∼ 3.8 (TGF-β1 expression increased ∼ 1.5 fold), whilst overexpression of PHB was able to increase levels ∼ 22 fold (TGF-β1 expression decreased by a factor of ∼ 2.5). Interestingly, overexpression of PHB was unable to reduce TGF-β1 levels to control levels and remained ∼ 2.1 times higher than control. This indicates that some pro-fibrotic capacity remained. The mRNA expression of PHB and TGF-β1 in the control viruses group was similar to the H-R group, which shows that the transduction with the vector did not affect the result.

On Western blot, protein expression levels closely followed the results of the mRNA experiments (Fig. 3). In general, this indicates that no alternate mechanisms were involved and that the gene silencing was effective in the current experimental approach. PHB expression under oxidative stress resulted in a ∼ 2.4 fold reduction with ∼ 42% remaining compared with control, and a 7.4 fold reduction in the H-R PHB^–^ group with ∼ 13% remaining compared with control (∼3.1 fold reduction compared with H-R). Conversely, PHB overexpression increased levels ∼ 1.7 fold compared with control and ∼ 4 fold compared with H-R. Similar to the mRNA results, TGF-β1 expression increased ∼ 3.4 fold compared with control and a further ∼ 1.6 fold under PHB knockdown conditions compared with H-R. However, overexpression of PHB was unable to reduce TGF-β1 protein levels to control levels, which remained ∼ 2.1 times higher than control. Changes in Col-IV and FN were in the same order of magnitude as TGF-β1. As observed from the TGF-β1 mRNA and protein levels, some pro-fibrotic capacity remained even under PHB overexpression, and figure 3A clearly shows that components involved in ECM deposition and RIF, Col IV and FN, remained ∼ 1.6 fold and ∼ 2.4 fold higher than control levels. Collectively, these results corroborate the mRNA expression experiments and show that PHB levels directly influence the fibrotic process, at least in a model cell system, but PHB overexpression was concurrently unable to reduce pro-fibrotic components to normoxic control levels.

### Redox status measurements

**Figure 4:**
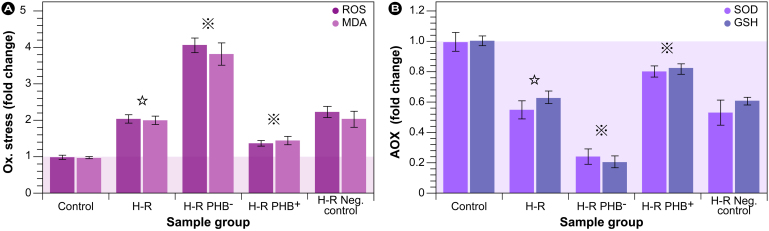
Assessment of oxidative stress (A) and antioxidative capacity (B). Changes in general reactive oxygen species (ROS) levels and malondialdehyde (MDA) generation, a final degradation product of lipid peroxidation, are shown in (A), and superoxide dismutase (SOD) and reduced glutathione (GSH) in (B). 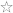: *P* < 0.01 compared with normal control group, 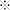: *P* < 0.01 compared with H-R group (cell injury induced by hypoxia reoxygenation group). H-R PHB^–^: transduced with lentivirus carrying siRNA PHB; H-R PHB^+^: transduced with lentivirus carrying *Phb*; H-R negative control: transduced with control viruses. AOX: antioxidant.

In order to assess the effect of PHB on the extent of intracellular oxidative stress and antioxidant status, indicators of these were evaluated in the five RTEC groups. As shown in figure 4, H-R induced a doubling of the ROS and MDA levels (Fig. 4A) and concomitantly a ∼ 1.7 fold reduction in SOD and GSH (Fig. 4B) compared with control. PHB knockdown resulted in an additional ROS/MDA level doubling compared with H-R only (∼4 fold compared with control) and a ∼ 2−3 fold reduction in endogenous antioxidants compared with H-R (∼1.7 fold compared with control). SOD levels were compromised, which consequently lowered the cell's capability to dismutate the superoxide anion, which in turn had consequences for mitochondrial stability (Fig. 1). Interestingly, PHB depletion had a major impact on lipid peroxidation relative to the total ROS levels, which shows that biomembranes are prime targets during fibrotic processes. However, figure 4 also shows that PHB overexpression was unable to fully ameliorate oxidative stress under H-R conditions compared with control, which is in general accord with the results in figure 3 and a still increased profibrotic capacity compared with control.

### Mitochondrial membrane potential and cell apoptosis

**Figure 5:**
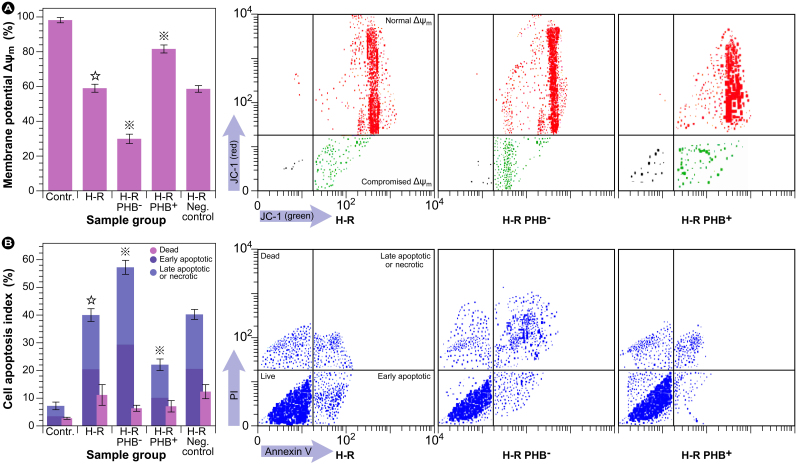
Flow cytometric assessment of (A) changes in the mitochondrial membrane potential (Δψ_m_) and (B) the induction of cell apoptosis in renal tubular epithelial cells (RTEC). Representative dot plots of JC-1 aggregates (red fluorescent) versus JC-1 monomers (green fluorescent) and staining of apoptotic cells with annexin V (cell surface phosphatidylserine detection) in response to various treatments. 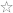: *P* < 0.01 compared with normal control group, 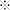: *P* < 0.01 compared with H-R group (cell injury induced by hypoxia reoxygenation group). PHB^–^: transduced with lentivirus carrying siRNA PHB; PHB^+^: transduced with lentivirus carrying *Phb*; Negative control: transduced with control viruses.

Since the redox equilibrium was compromised by H-R and the microscopic evaluation of the cells in figure 1 both showed that attenuation of PHB expression led to increased cellular damage and loss of mitochondrial integrity, the impact on mitochondrial functionality and resulting cell death was further evaluated via flow cytometry. A distinctive feature of early stages of apoptosis is the derangement of mitochondrial function, which includes changes in the mitochondrial membrane potential (Δψ_m_). These are assumed to be induced through opening of the mitochondrial permeability transition pore (MPTP), thereby allowing passage of ions and small molecules, which in turn causes decoupling of the respiratory chain and release of cytochrome c into the cytosol. In RTEC cells, clearly a collapse in membrane potential (Δψ_m_) due to H-R occurred (∼1.7 fold compared with control), which doubled (∼1.9 fold compared with H-R) during attenuation of PHB expression (∼3.3 fold compared with control), since the number of cells with functional Δψ_m_ decreased progressively (Fig. 5A). At the same time, the percentage total apoptotic cells increased from an initial 7.2 ± 1.2% in control cells to 40 ± 2.1 as a result of H-R, and 57.1 ± 2.8% when PHB was additionally depleted (Fig 5B). Even though PHB overexpression reduced the number of apoptotic cells ∼ 1.8 fold compared with H-R, overexpression was unable to reduce apoptosis to control levels (still ∼ 3.1 fold higher than control), which was comparable to the results of the oxidative stress measurements. Furthermore, neither the induced H-R, nor the knockdown of PHB seemed to significantly influence the distribution between early and late apoptosis.

### Correlation analysis

Correlation analysis showed that PHB protein levels were negatively correlated with ROS, MDA, TGF-βl, Col-IV, FN, cell apoptosis index (*r* = −0.895, −0.764, −0.798, −0.826, −0.817, −0.735; each *P* < 0.01), but positively correlated with SOD, GSH and mitochondrial membrane potential (*r* = 0.807, 0.815, 0.739; each *P* < 0.01). Overall, all experiments and the correlation analysis suggest that PHB at least partially counteracts oxidative stress and RIF in a model cell system.

## Discussion

Renal oxidative stress is considered a fundamental factor that contributes to the development of tubular atrophy and interstitial fibrosis, which in turn are both manifestations of renal disease progression and ultimately lead to renal failure^15^,^16^,^17^. Several groups report that expression of profibrotic factors or proteins involved in ECM accumulation are induced under hypoxia/reperfusion. Hung et al^18^ found that hypoxic conditions induced the elevated secretion of TGF-βl by mesenchymal stem cells and promoted the growth, motility and invasion ability of breast cancer cells. Fuchshofer and co-workers^19^ reported that hypoxia in the retinal pigment epithelium might lead to an increase of Col-IV and FN deposition. Jin et al.^20^ demonstrated that hypoxia treatment could increase the mitochondrial membrane potential and cell apoptosis in H9c2 cells. Furthermore, PHB, pleiotropic protein involved in various cellular functions, including proliferation, tumour suppression, apoptosis, transcription, and mitochondrial protein folding, was postulated to be involved in RIF. Indeed, we recently showed that prohibitin downregulation *per se* increased RIF in unilaterally ureteral obstructed rats^10^. However, whether reduced PHB expression levels should be considered a risk factor for RIF or if approaches that increase PHB might present a possible therapeutic option, it remains to be established.

In this investigation, we studied if PHB expression modulation was able to affect components involved in fibrosis, oxidative stress, and apoptosis as the final detrimental outcome of RIF in a RTEC cell model. Our experimental approach was based on the endogenous induction of oxidative stress through hypoxia/reoxygenation, which is considered a physiologically relevant method for simulating the conditions found in RIF in the intact organism. Modulation of PHB expression was achieved by either transducing cells with a lentivirus vector carrying an interfering RNA to downregulate PHB expression or by overexpressing PHB with a lentivirus vector carrying *Phb* under H-R conditions.

**Figure 6:**
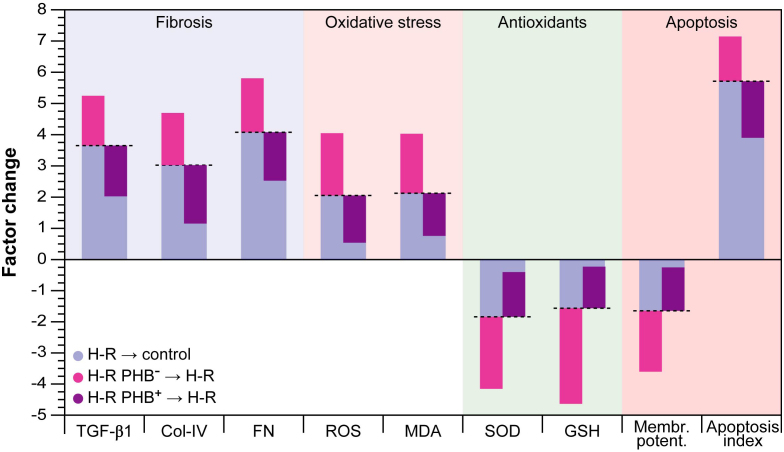
Overview of the changes induced by hypoxia/reoxygenation (H-R) and prohibitin (PHB) expression modulation under H-R conditions. Negative signs indicate factor decrease. Note that the blue bars show the change relative to normoxic control cells, whereas all other bars show the change relative to H-R. H-R PHB^–^: transduced with lentivirus carrying siRNA PHB; H-R PHB^+^: transduced with lentivirus carrying *Phb*.

Our experimental results show that ROS and MDA, as indicators of oxidative stress, were increased two-fold, and that SOD and GSH, as markers for the antioxidative capacity, were concomitantly and in the same order of magnitude reduced in the H-R group compared with control (Fig. 6; blue bars). This indicated that H-R induced endogenous oxidative stress in this model system and morphological assessment via microscopy confirmed the presence of typical signs thereof, i.e., membrane blebbing, diffuse intracellular appearance, and mitochondrial swelling and loss of integrity. These results are in conformity with the large body of evidence reported in the literature for a myriad of cell systems. Furthermore, PHB expression was reduced ∼ 2.5 fold in the H-R group compared with the control group. Concomitantly, the expression of TGF-βl, Col-IV, and FN in the H-R group was up-regulated by at least a factor of three. Additionally, a clear collapse in mitochondrial membrane potential (Δψ_m_) and an increase in cell apoptosis rate in the H-R group were observed compared with the control group. These results are in conformity with our previous work in which we established that unilateral ureteral obstruction increased RIF and concomitantly caspase-3 expression and apoptosis in rats^10^. It is therefore reasonable to assume that PHB might act as an antioxidant, or at least induce protective mechanisms that reduce oxidative stress in RTEC cells, including maintaining mitochondrial stability. Recent work by Liu et al.^21^ showed that PHB acts as a chaperone in myocardial cells and stabilizes respiratory proteins via a ring-like structure with 16–20 alternating PHB1 and PHB2 subunits^22^.

Subsequent interference with the expression levels under H-R showed that reduction of PHB protein expression by gene silencing resulted in a further increase in ROS and MDA levels on the one hand, and a reduction in SOD and GSH on the other (Fig. 6); reduction of GSH was more pronounced compared with SOD. Furthermore, measurement of Δψ_m_ and cell apoptosis rate showed that this condition further promoted mitochondrial membrane depolarization and increased apoptosis. Morphological assessment via optical and electron microscopy confirmed changes in the general cell and mitochondrial morphology as the overall appearance was significantly worsened compared with H-R (Fig. 1). Conversely, overexpression of PHB via lentivirus transfection with *Phb* distinctly reduced ROS and MDA and increased SOD and GSH. Since GSH is such a potent antioxidant, the observation that a significant restoration of its levels by PHB overexpression was achievable is (patho)biologically highly relevant. Reduction of PHB levels further increased expression of TGF-βl, Col-IV, and FN at least 1.5–2.4 fold compared with H-R, and therefore PHB depletion might aggravate ECM deposition and fibrosis in the intact hypoxic kidney.

The observations in this study were largely in agreement with results reported by others. For instance, Muraguchi et al.^23^ reported that overexpression of PHB prevented mitochondrial membrane depolarization, and inhibited cell death induced by hypoxia in H9c2 cardiomyocytes. Liu et al.^21^ reported that, when compared with untransfected cardiomyocytes, PHB overexpression protected mitochondria from hydrogen peroxide-induced oxidative injury, and concurrently suppressed the mitochondria-mediated apoptosis pathway and reduced the change in mitochondrial membrane permeability transition. Kathiria and co-workers^24^ described that gene silencing of PHB in intestinal epithelial cells induced mitochondrial autophagy via increased intracellular ROS. Inhibition of autophagy during PHB knockdown exacerbated mitochondrial depolarization and reduced cell viability. The work by P. Zhou et al.^25^ revealed that upregulation of PHB in neuronal cultures or hippocampal slices exerted neuroprotective effects, whereas PHB gene silencing increased neuronal vulnerability; an effect associated with loss of mitochondrial membrane potential and increased mitochondrial ROS production.

In conclusion, we show that an increased PHB expression downregulates ROS and MDA levels, and increases the expression of SOD and GSH levels *in vitro*. Concomitantly, TGF-βl, Col-IV, and FN expression is downregulated and consequently ECM deposition is reduced. Furthermore, this study is also the first to show that an increase in PHB reduces RTEC apoptosis *in vitro*. PHB therefore acts as a positive regulator of the mechanisms that counteract oxidative stress and ECM component accumulation in RTEC cells in culture and reduced PHB levels might represent a risk factor for RIF in the intact organ. Currently, we are investigating if these results can be confirmed in a more physiologically relevant setting of renal interstitial fibrosis, i.e. unilateral ureteral obstruction in rats, which is a well-characterized model for experimental obstructive nephropathy.

## Methods

### RTEC cell culture, induction of oxidative stress, and gene interference

The rat renal proximal tubular epithelial cell line (RTEC), NRK-52E, was purchased from the Cell Bank and Type Culture Collection of the Chinese Academy of Sciences (Shanghai Institute of Cell Biology, Shanghai, China) and maintained in DMEM (2 mM glutamine, 1% non-essential amino acids) supplemented with 5% foetal bovine serum (FBS) in a humidified atmosphere (37°C; 5% CO_2_). Oxidative stress and cell injury was induced by hypoxia/reoxygenation (H-R) as described in more detail by Bhogal *et al.*^14^. Briefly, to determine the effect of PHB expression modulation, cells were divided into five groups: (i) normoxic RTEC control (control), (ii) untransduced cells subjected to H-R (H-R), (iii) cells transduced with lentivirus carrying PHB-siRNA subjected to H-R (H-R PHB^–^), (iv) cells transduced with lentivirus carrying *Phb* subjected to H-R (H-R PHB^+^), and (v) cells transduced with control viruses subjected to H-R (H-R negative control); *n* = 6. Cells in the H-R PHB^–^, H-R PHB^+^, and H-R negative control groups were subjected to 48 h gene interference, overexpression, or mock treatment. The concentration of the lentivirus vector was 2.5 × 10^6^ TU/ml and 400 μg/ml hexadimethrine bromide (Polybrene©) was added to enhance the lentivirus infection ratio (final concentration was 8 μg/ml). The lentivirus vectors carrying PHB-siRNA or *Phb* were designed and custom made by Invitrogen (Invitrogen/Life Technologies, Grand Island, NY, USA) based on the ViraPower™ pLP1 (8889 bp) vector. Subsequently, hypoxia was induced by placing the cells in an airtight incubator (RS Mini Galaxy A incubator, Wolf Laboratories, UK), flushed with 5% CO_2_ and 95% N_2_ until the O_2_ content reached 0.1%. After 24 h hypoxia, the medium was aspirated and replaced with fresh oxygenated medium (37°C), and the cells were returned to normoxic conditions for 2 h.

### Microscopic evaluation of cellular and sub-cellular morphology in RTEC cells

Initial evaluation of RTEC morphology was performed via optical microscopy on an inverted phase-contrast microscope (Olympus, Co., Tokyo, Japan).

Detailed and high-resolution observations were performed with scanning electron microscopy (SEM). Samples were prefixed by immersion in 2% glutaraldehyde in 0.1 M phosphate buffer and post-fixed for 2 h in 1% osmic acid dissolved in PBS. Samples were treated in a graded series of ethanol and *t*-butyl alcohol, dried in a model ES-2030 freeze dryer (Hitachi, Tokyo, Japan), platinum-coated using an IB-5 ion coater (Eiko, Kanagawa, Japan), and observed using a VEGA3 SEM system (Tescan, a.s., Brno, Czech Republic).

Mitochondrial morphological changes were imaged with transmission electron microscopy (TEM). Cells were centrifuged and the pellet was loaded on aluminum discs with 100 mm depth (Engineering Office M. Wohlwend GmbH, Sennwald, Switzerland) and covered with a flat disc. The sandwiched sample was frozen in a HPM010 high-pressure freezing machine (Leica Microsystems/Bal-Tec, Liechtenstein). Cells were subsequently freeze-substituted in a AFS2 freeze substitution device (Leica Microsystems, Vienna, Austria) in anhydrous acetone containing 2% glutaraldehyde and 0.2% tannic acid/osmium tetroxide for 3 days at –90°C and then warmed up to –30°C over 24 h. At ambient temperature, samples were washed three times with acetone, incubated for 1 h with 2% osmium tetroxide, washed once more three times with acetone, and incubated for 5–7 days in a series of increasing concentrations of Epon in acetone. After polymerization at 60°C, 60–80 nm sections were stained with uranyl acetate and lead citrate and examined with an H-7650 transmission electron microscope (Hitachi, Chula Vista, CA, USA) operating at an accelerating voltage of 120 kV.

### Real time reverse transcription polymerase chain reaction to detect PHB and TGF-β1 mRNA expression

Total RNA of RTEC was extracted with TRIzol (Life Technologies-Invitrogen, Grand Island, NY, USA). Signals was measured with a Gel Doc XR+, UV/*vis*-Molecular Imager (Bio-Rad Laboratories, Hercules, CA, USA) and evaluation of the 18S and 28S RNA bands after agarose gel electrophoresis confirmed that there had been no RNA degradation^26^. Primers were designed according to standard primer design principles with Primer Premier 5.0 (Premier Biosoft, Palo Alto, CA, USA): PHB = F 5'–TGGCGTTAGCGGTTACAGGAG–3' and R 5'-GAGGATGCGTAGTGTGATGT TGAC-3'; TGF-β1 = F 5'-TGAGCACTGAAGCGAAAGCC-3' and R 5'-GATTCAA GTCAACTGTGGAGCAAC-3'; β-actin = F 5'-GCCCCTGAGGAGCACCCTGT-3' and R 5'-ACGCTCGGTCAGGATCTTCA-3'.

One microgram total RNA was reverse transcribed into cDNA with an ExScript RT reagent kit (Thermo Scientific-Fermentas, Waltham, MA, USA). PHB and TGF-β1 were amplified with SYBR Premix Ex Taq (Roche Inc., Basel, Switzerland). As an internal loading control, gene expression of the housekeeping β-actin (*ACTB*) was used as an internal loading control and to determine reverse transcription efficiency. The average threshold cycle (Ct; the cycles of template amplification to the threshold) was determined for each sample and the. fold change in the data was analyzed according to^27^.

### Western-blot analysis

Proteins were isolated from homogenized RTEC cells with radio immunoprecipitation assay (RIPA) lysis buffer (Sigma-Aldrich Corp., St. Louis, MO, USA) containing 0.25 nM of the protease inhibitor phenylmethanesulfonyl fluoride (PMSF; Sigma-Aldrich Corp., St. Louis, MO, USA). After protein concentration, quantization was performed with the modified Bradford assay (Bio-Rad Laboratories, Hercules, CA, USA)^28^ and 40 mg total protein was subsequently used for Western blotting with primary antibodies against PHB (1∶2000), TGF-β1 (1∶1500), Col-IV (1∶2000), and FN (1∶2000); β-actin was used as an internal loading control (all antibodies were purchased from Abcam, Cambridge, MA, USA). Near-infra red fluorescence from manually selected bands of interest were imaged with an Odyssey Fc scanner (Li-Cor, Lincoln, NE, USA); Raw fluorescence intensities were background subtracted (intra-lane) using Li-Cor Odyssey 3.0 analytical software^29^.

### Flow cytrometric evaluation of mitochondrial membrane potential and cellular apoptosis in RTEC cells

The change in mitochondrial membrane potential (Δψ_m_) was quantified with a JC-1 kit according to the manufacturer's protocol (Agilent Technologies/Stratagene, La Jolla, CA, USA). JC-1 (5,5′, 6,6′-tetrachloro-1,1′,3,3′-tetraethyl benzimidazolylcarbocyanine iodide) is a lipophylic cationic fluorescent compound that accumulates in the mitochondrial matrix of intact mitochondria with large Δψ_m_ to form red emitting (585 nm) J-aggregates. During membrane depolarization (apoptosis), JC-1 cannot accumulate in the mitochondrial matrix, but remains in the cytosol, where the monomers emit bright green fluorescence at 530 nm. Cells were harvested, washed, and stained with JC-1 for 15 min at 37°C. Cells were then washed with PBS and Δψ_m_ was measured by flow cytometry on a BD FACScalibur (BD Biosciences, San Jose, CA, USA)^30^. The ratio of red (585 nm) to green (480 nm) reflects the mitochondrial membrane potential.

Apoptotic cells were detected by staining with an annexin V-FITC apoptosis detection kit (Sigma-Aldrich Corp., St. Louis, MO, USA) according to the manufacturer' protocol. Briefly, RTEC cells were washed with PBS, trypsinated with trypsin-EDTA, collected in growth medium/serum, and the cell suspension was centrifuged at 1000 × rpm for 5 min. Subsequently, the cells were resuspended in annexin V binding buffer, and incubated with propidium iodide (PI) and annexin V-FITC, and for 15 min at room temperature^31^. Apoptotic cells were identified and quantified using an EPICS XL-MCL flow cytometer (Beckman Coulter, Miami, FL, USA).

### ROS, Lipid peroxidation, and antioxidant measurements

ROS measurements were essentially performed according to Hempel *et al*.^32^, with some modifications. Cell supernatants were obtained by scraping cell monolayers, subsequent sonication and centrifugation (15000 × g; 5 min) at 4°C, and 500 μl supernatant was incubated for 3 h at 37°C with 10 μL of a 10 μM DCF-DA (2,7-dichlorodihydro-fluorescein diacetate) solution (Life Technologies/Molecular probes, Eugene, OR, USA). The fluorescence signal was measured at 485/525 nm on a S-3100 spectrofluorometer (Scinco Co. Ltd., Seoul, Korea), equipped with a 1024 channel photodiode array detector and expressed as arbitrary units.

Malonyldialdehyde (MDA), glutathione (GSH), and superoxide dismutase (SOD) were determined in cell supernatants obtained by scraping cell monolayers, and subsequent sonication and centrifugation (15000 × g; 5 min) at 4°C. Protein concentration of samples was determined with BCA (bicinchoninic acid) protein assay kit (Sigma-Aldrich Corp., St. Louis, MO, USA) with BSA as a standard. Absorbance was measure spectrophotometrically at 562 nm.

MDA was determined spectrophotometrically using the N-methyl-2-phenylindole (NMPI) method according to Bergman et al.^33^. Briefly, 50 μL sample or tetramethoxy propane (standard; 0.8–8 μM) was mixed with 160 μL of a 10 mM NMPI solution and 40 μL methanesulfonic acid (15.4 M), and subsequently incubated for 48 min at 45°C. The absorbance of the chromophore was measured at 586 nm.

SOD activity was determined by assessing the inhibitory effect of SOD on the reduction of nitroblue tetrazolium (NBT) by the superoxide anion generated by the xanthine/xanthine oxidase system according to Kasemsri and Armstead^34^. The absorbance was measured at 560 nm.

GSH levels were determined spectrophotometrically at 405 nm via the modified 5,5′-Dithiobis(2-nitrobenzoic acid) glutathione disulfide (DTNB-GSSG) reductase recycling method described by Anderson^35^.

### Statistical analysis

All data are shown as mean ± standard deviation (SD). To compare the groups in relation to parameters with normal distribution, one-way analysis of variation (ANOVA) with post-hoc Fisher's LSD (least significant difference) was used. Conversely, for those parameters without normal distribution, Kruskal-Wallis with post-hoc Mann-Whitney (only for the weight parameter) was used. Pearson's correlation coefficients were used to determine the relationships between the indicators for detection in the cell culture. A value of P < 0.05 was accepted as statistically significant. Statistical analysis was performed using the Statistical Package for Social Sciences–SPSS, version 13.0 (SPSS Inc., Chicago, IL, USA).

## Author Contributions

T.B.Z., F.Y.L. and W.F.H. performed experiments. T.B.Z. initiated and designed the study. T.B.Z., G.P.C.D. and Y.H.Q. analyzed data, critically appraised the study and supervised the experiments. Y.H.Q. provided chemicals and equipment. T.B.Z. and G.P.C.D. made the figures and wrote and revised the manuscript. All authors read and approved the final manuscript.
